# Human iPSCs from Aged Donors Retain Their Mitochondrial Aging Signature

**DOI:** 10.3390/ijms252011199

**Published:** 2024-10-18

**Authors:** Imane Lejri, Zameel Cader, Amandine Grimm, Anne Eckert

**Affiliations:** 1 Research Cluster Molecular and Cognitive Neurosciences, University of Basel, 4002 Basel, Switzerland; imane.lejri@upk.ch (I.L.); amandine.grimm@unibas.ch (A.G.); 2 Neurobiology Lab for Brain Aging and Mental Health, University Psychiatric Clinics Basel, 4002 Basel, Switzerland; 3 Translational Molecular Neuroscience Group, Nuffield Department of Clinical Neurosciences, Weatherall Institute of Molecular Medicine, University of Oxford, Oxford OX3 9DS, UK; zameel.cader@ndcn.ox.ac.uk; 4 Department of Biomedicine, University of Basel, 4055 Basel, Switzerland

**Keywords:** aging, bioenergetics, human-induced pluripotent stem cells, mitochondria, mitochondrial morphology, oxidative stress

## Abstract

Aging represents the leading risk factor for developing neurodegenerative disorders. One of the nine hallmarks of aging is mitochondrial dysfunction. Age-related mitochondrial alterations have been shown to affect mitochondrial energy metabolism, reduction-oxidation homeostasis, and mitochondrial dynamics. Previous reports have shown that induced pluripotent stem cells (iPSCs) from aged donors do not keep the aging signature at the transcriptomic level. However, not all aspects of aging have been investigated, and especially not the mitochondria-related aging signature. Therefore, the present study compared the mitochondrial function in iPSCs from healthy aged donors compared to those of young donors. We addressed whether aged iPSCs may be used as drug-screening models of “aging in a dish” to identify therapies alleviating mitochondria aging. Compared to iPSCs from young donors, we demonstrate that iPSCs from aged donors show impaired mitochondrial bioenergetics and exhibit a rise in reactive oxygen species generation. Furthermore, aged iPSCs present a lower mitochondrial mass and alterations in the morphology of the mitochondrial network when compared to iPSCs from young donors. This study provides the first evidence that the aging phenotype is present at the mitochondrial level in iPSCs from aged donors, ranging from bioenergetics to mitochondrial network morphology. This model might be used to screen mitochondria-targeting drugs to promote healthy aging at the mitochondrial level.

## 1. Introduction

In aging, several hallmarks are reported, including mitochondrial dysfunction, which is also known to be linked to age-related neurodegenerative disorders [1,2,3]. Age-related mitochondrial damage has been shown to affect mitochondrial energy metabolism and dynamics and to reduce-oxidation (redox) homeostasis [4]. Aging research tools include diverse organism models [5]. As mouse models have some limitations regarding translatability to human physiology and lifespan, advanced human in vitro models are needed [6]. Also, the inaccessibility of live human brain samples, such as neurons, has slowed progress in this area. Induced pluripotent stem cells (iPSCs) represent “synthetic” stem cells reprogrammed or induced from somatic cells cultured with various transcription factors such as Klf4,c-Myc, Oct4, and Sox2 [7]. The first human iPSCs were created from a biopsy of skin fibroblasts [8]. A number of biological correlates indicate that fibroblasts are an appropriate model for the study of aging at the cellular level. The recent development of technologies allows fibroblasts to be reprogrammed into iPSCs for neural cell generation or converted directly into neural cells (induced neurons, iNs). This presents an opportunity to study important aspects of the central nervous system function in vitro [7]. Recently, it has been shown that iPSCs present reversed age-associated molecular features, such as telomere lengthening and age-related changes in the transcriptome [9]. However, there were no reported data regarding the mitochondrial function, which is one of the nine hallmarks of aging, in iPSCs from healthy old donors. The iPSCs may retain the epigenetic characteristics of their tissue of origin, potentially impacting their differentiation ability [9]. Therefore, we wanted to investigate if iPSCs from healthy aged donors still show signs of aging at the mitochondrial level. We compared the mitochondrial bioenergetics and structure in human iPSCs from elderly donors to those from young donors. Our findings indicate that iPSCs from aged donors have lower adenosine triphosphate (ATP) synthesis, a decrease in mitochondrial membrane potential (MMP), higher production of mitochondrial reactive oxygen species (ROS), and defects in mitochondrial respiration and glycolytic function compared to iPSCs from young donors. Additionally, we observed changes in the shape of the mitochondrial network in aged iPSCs, resulting in more fragmented and shorter mitochondria.

## 2. Results

### 2.1. iPSCs from Aged Donors Display Mitochondrial Bioenergetic Deficits

**Figure 1:**
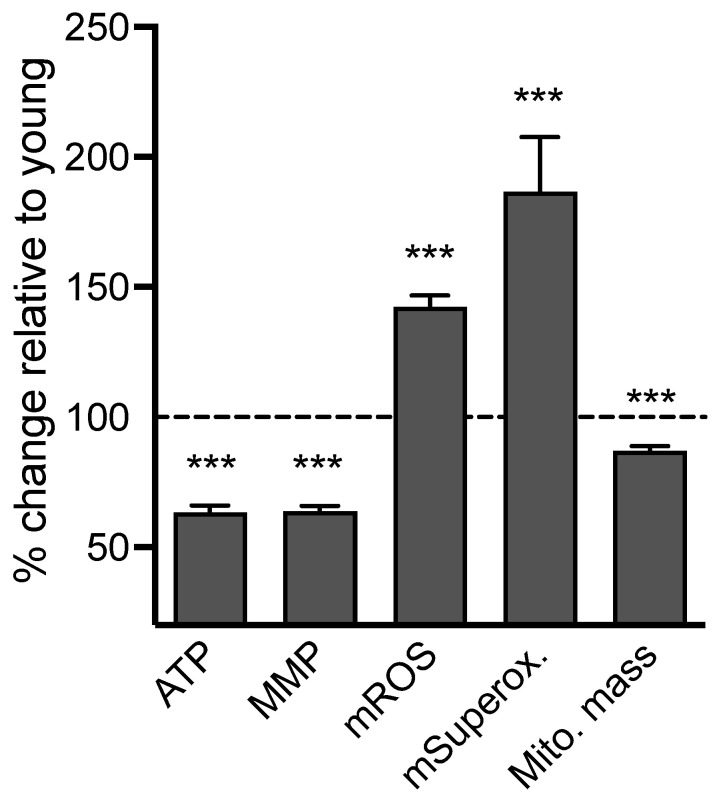
Bioenergetic readouts in iPSCs from young versus old donors. Readouts include ATP generation (ATP), mitochondrial membrane potential (MMP), mitochondrial ROS (DHR), mitochondrial superoxide anion (MitoSOX) levels, and mitochondrial mass. Data were normalized on the CellTracker blue signal, an indicator of living cell surface area, and are shown as the percentage of iPSCs derived from young donors. Values represent the mean ± SEM of 10–12 replicates/donor/readout, for four young and four aged donors, and three independent experiments (Total n = 123–150 replicates/group), and are shown as the percentage of iPSCs derived from young donors. (dashed line). Student *t*-test between iPSCs from young vs. aged donors *** *p* < 0.001.

First, we evaluated the total ATP level, mitochondrial membrane potential (MMP), reactive oxygen species (ROS) levels, and mitochondrial mass in iPSCs obtained from both young and old donors in order to understand their bioenergetic characteristics (see Figure 1). In the iPSCs obtained from old donors, ATP and MMP levels were significantly reduced by 36.8% and 36.25%, respectively, compared to iPSCs from young donors (see Figure 1). Additionally, the fluorescence levels of the mitochondrial reactive oxygen species (mROS) indicator dihydrorhodamine 123 and the mitochondrial superoxide anion (mSuperox.) indicator MitoSOX were significantly higher in old donor iPSCs compared to young donor iPSCs (see Figure 1).

Because these findings might be linked to changes in mitochondrial mass in iPSCs from aged donors, the MitoTracker Green FM (MTG), an MMP-independent mitochondrial dye, was used to assess this parameter [10]. A decrease in the MTG fluorescence signal was observed in iPSCs from elderly donors compared to iPSCs from young donors (Figure 1, Supplementary Figure S1). Specifically, there was a 13% decrease in the MTG signal in iPSCs from aged donors compared to young donors, indicating lower mitochondrial content in aged cells. To determine whether the difference in mitochondrial mass between young and aged donors affects parameters such as the MMP or mitochondrial ROS level, these parameters were normalized to the MTG signal (Supplementary Figure S2). Even after normalization, a significant difference was still observed between young and aged iPSCs, with a 26.8% decrease in MMP, a 63% increase in mitochondrial ROS, and a 114% increase in mitochondrial superoxide anion radicals in cells from aged donors compared to young ones. Overall, these findings suggest that iPSCs from aged donors exhibit bioenergetic impairments when compared to those from young donors.

### 2.2. Decrease in Mitochondrial Respiration and Increase in Glycolytic Function in iPSCs from Aged Donors Compared to Young Donors

**Figure 2:**
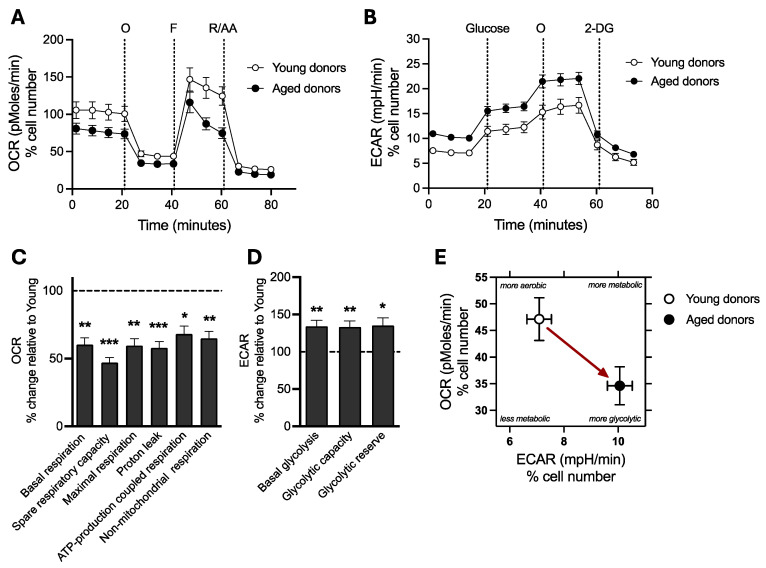
Analysis of mitochondrial respiration and cellular glycolysis in young versus old iPSCs. (**A**) oxygen consumption rate (OCR) and (**B**) extracellular acidification rate (ECAR) were measured before and after the sequential injection of specific modulators (A: Mito Stress test and B: Glycolysis stress test) using the Seahorse XF HS Mini Analyser. (**C**) OCR-based respiratory parameters, including basal respiration, spare respiratory capacity, maximal respiration, proton leak, ATP-production coupled respiration, and non-mitochondrial oxygen consumption, were calculated in young and aged iPSCs. (**D**) ECAR-based glycolysis parameters, including basal glycolysis, glycolytic capacity, and glycolytic reserve, were calculated in young and aged iPSCs. (**E**) Representative bioenergetics phenotype of young and aged iPSCs is depicted as the mean of the basal ECAR on the abscissa and the mean of the basal OCR on the ordinate. The red arrow indicates a metabolic shift from a more aerobic phenotype to a more glycolytic phenotype between young and aged cells. (**A**–**E**) All the data were normalized on the CellTracker blue signal, an indicator of living cell surface area. (**C**,**D**) Values represent the mean ± SEM of three independent tests (n = 15–17 replicates per condition for OCR evaluation, n = 24–30 replicates for ECAR evaluation, four human iPSCs from young donors or four human iPSCs from aged donors per group and are shown as the percentage of iPSCs derived from young donors. (dashed line). * *p* < 0.05, ** *p* < 0.01, *** *p* < 0.001, Student *t*-test between iPSCs from young donors vs. iPSCs from aged donors. AA: antimycin A, 2-DG: 2-deoxyglucose, ECAR: Extracellular acidification rate, F: FCCP, OCR: Oxygen Consumption Rate, O: oligomycin, R: rotenone.

Two main pathways, mitochondrial oxidative phosphorylation (OxPhos) and cellular glycolysis, generate ATP molecules. Oxygen consumption rate (OCR) is an indicator of mitochondrial respiration (Figure 2A), while the extracellular acidification rate (ECAR) is an indicator of cellular glycolysis (Figure 2B). Both were monitored separately in real time using the Seahorse XF Analyzer. A significant reduction in the OCR was observed in iPSCs from aged donors compared to iPSCs from young donors (Figure 2A), while the ECAR was increased (Figure 2B). The sequential injection of the respiratory inhibitor oligomycin (O), FCCP, and rotenone/antimycine A (R/AA) allowed for the determination of specific respiratory parameters (Figure 2A,C). Namely, we observed that iPSCs from aged donors presented a significant decrease in basal respiration (−39.8%), spare respiratory capacity (−53%), maximal respiration (−40.6%), proton leak (−42.2%), ATP-production coupled respiration (−31.9%), and non-mitochondrial respiration (−35.1%) when compared to iPSCs from young donors (Figure 2C). This indicates mitochondrial metabolic dysfunction in cells from aged donors. Similarly, the sequential injection of glucose, oligomycin, and 2-deoxyglucose (2-DG) allowed for the determination of specific glycolysis parameters (Figure 2B,D). Notably, we observed that iPSCs from aged donors presented increased basal glycolysis (+34%), glycolytic capacity (+33%), and glycolytic reserve levels (+35%) when compared to iPSCs from young donors (Figure 2D). The bioenergetic profile showing the basal OCR and ECAR of young and aged iPSCs indicates that young iPSCs are more aerobic and less glycolytic (Figure 2E). In contrast, aged iPSCs are more glycolytic and less aerobic, suggesting a metabolic shift from OxPhos to glycolysis.

These data show that iPSCs from aged donors exhibit decreased mitochondrial respiration and increased glycolytic activity, possibly as a compensatory mechanism to meet the cells’ bioenergetic demands.

### 2.3. Age-Related Modification of the Mitochondrial Network Morphology

**Figure 3:**
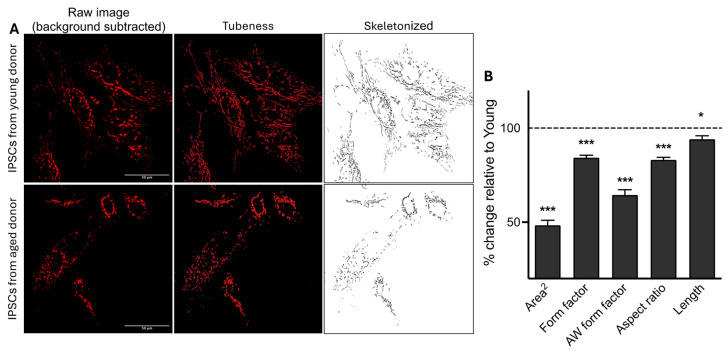
Impact of age on the mitochondrial network morphology in iPSCs. (**A**) Representative z-projection microscopy images of the mitochondrial network stained with the CMXROS Mitotracker Red in iPSCs from young and aged donors (×94.5 magnification). The left panels display the raw images after background subtraction (rolling ball radius = 20 pixels). The middle panels (tubeness) display the same pictures after processing the morphometry macro on the FIJI software. The right panels (skeletonized) present binary images of the mitochondrial network (in black) after further image processing in FIJI using the “skeletonize” function. (**B**) Quantification of mitochondrial network morphology parameters, including the area^2^ (average of the size of mitochondria), form factor (elongation of mitochondria), area-weighted (AW) form factor (a variant of form factor with a bias towards larger mitochondria), aspect ratio (branching), and mitochondria length in iPSCs from young and aged donors. Values represent the mean ± SEM of three independent experiments (n = 85–90 replicates per condition, four human iPSCs from young donors or four human iPSCs from aged donord per group), and are shown as the percentage of iPSCs derived from young donors (dashed line). * *p* < 0.05, *** *p* < 0.001, Student *t*-test iPSCs from young donors vs. iPSCs from aged donors.

Changes in the bioenergetic phenotype are closely linked to alterations in the mitochondrial morphology [11]. A lower bioenergetic status often correlates with fragmented mitochondria, and higher bioenergetic conditions are associated with more elongated mitochondria [12,13]. Since iPSCs from aged donors exhibited bioenergetic deficiencies, we proceeded to examine the effects of aging on the mitochondrial network morphology using fluorescence microscopy. Mitochondria were stained using the mitochondrial dye CMXROS MitotrackerRed for visualization. Subsequently, the images were analyzed to evaluate the morphology of the mitochondria using FIJI software (Figure 3A). All the mitochondrial network morphology parameters were significantly altered in aged iPSCs (Figure 3B). We observed a decrease in the area^2^ (−51%), the form factor (−15.8%), the area-weighted form factor (−35.6%), and the aspect ratio (−17%) in iPSCs from aged donors compared to iPSCs from young donors.

Additionally, we observed a significant decrease in mitochondrial length (Figure 3B) in iPSCs from older donors compared to those from young donors. Thus, iPSCs from aged donors exhibited more fragmented mitochondria, as indicated by a lower “form factor” value and a shorter length. In contrast, iPSCs from young donors were more tubular, with higher form factor values and longer mitochondria.

Overall, our data indicates an age-related impact on mitochondrial network morphology in iPSCs. Specifically, iPSCs from aged donors exhibit less elongated and more fragmented mitochondria.

### 2.4. Correlations Between Bioenergetic Parameters and Donors’ Ages

To further apprehend the link between mitochondrial impairments and aging in iPSCs, Pearson’s linear regression analysis was performed with data from each donor (Supplementary Figure S3). We observed an age-related decrease in ATP level (R^2^ = 0.41, *p* = 0.08), in MMP (R^2^ = 0.44, *p* = 0.06), and in basal OCR (R^2^ = 0.45, *p* = 0.06), as well as a significant age-related increase in basal ECAR (R^2^ = 0.61, *p* = 0.022). Also, the age-related decrease in ATP level was linked to the age-related decrease in MMP (R^2^ = 0.61, *p* = 0.06) and significantly correlated with mitochondrial elongation (R^2^ = 0.59, *p* = 0.025). These data suggest that the decline in ATP levels with increasing age is parallel with a reduction of MMP and an increase in mitochondrial fragmentation. We also observed a significant and negative correlation between the basal OCR and ECAR (R^2^ = 0.61, *p* = 0.022), suggesting again an age-related shift from OxPhos to glycolysis to sustain cells’ energy needs during aging.

## 3. Discussion

Studies have focused mainly on age-related dysfunction in fibroblasts, but the characterization of mitochondrial impairment in human iPSCs from healthy elderly donors has not been explored [14,15,16]. Although reprogramming towards the pluripotency stage has been shown to reset cellular clock aging and remove most of the cellular hallmarks associated with aging, a partial representation might be possible. Therefore, our study aimed to evaluate whether iPSCs from healthy elderly donors still show an aging signature at the mitochondrial level. We found that iPSCs from elderly donors exhibited the following when compared to iPSCs from young donors:

1. Lower levels of ATP and MMP, as well as reduced mitochondrial mass accompanied by increased ROS production;
2. Decreased mitochondrial respiration and metabolic alterations, including heightened glycolytic activity;
3. Disturbances in mitochondrial network morphology resulting in mitochondrial fragmentation.

**Figure 4:**
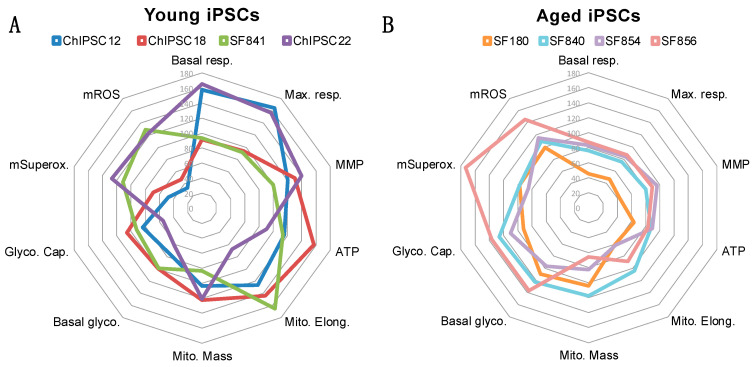
Radar plots summarizing the key data from each young and aged donor. (**A**) Summary of data obtained in iPSCs derived from four young donors. (**B**) Summary of data obtained in iPSCs derived from four aged donors. The radar plots were generated with Excel. The data used to create the radar plot are represented as a percentage of the mean young (**A**) or as a percentage of the mean aged (**B**). Basal glyco.: basal glycolysis; Basal resp.: basal respiration; Glyco. Cap.: Glycolytic capacity; Max. resp.: Maximal respiration; Mito. Elong.: Mitochondrial elongation; Mito. Mass: mitochondrial mass; MMP: mitochondrial membrane potential; mROS: mitochondrial ROS; mSuperox.: mitochondrial superoxide anion radicals.

Figure 4 summarizes key data obtained in the iPSCs from the four young donors (Figure 4A) and the four aged donors (Figure 4B). Even if interindividual differences can be observed, young donors clearly present higher mitochondrial bioenergetic activity (e.g., OxPhos, ATP, MMP), while aged donors show a decrease in mitochondrial bioenergetics and an increase in ROS production and glycolytic activity.

The physiological aging process begins after the age of 60, and aging is the primary risk factor for the onset of most neurodegenerative diseases [17], including Alzheimer’s disease (AD) and Parkinson’s disease (PD) [18]. The neurological burden occurs between the ages of 60 and 84 years [19], and the prevalence of most of the neurological conditions that cause disability rises sharply with age [20].

The free radicals theory of aging posits that mitochondria are central to the process, serving as both a source and target of reactive oxygen species (ROS) [4]. As we age, oxidative stress increases, potentially damaging proteins, lipids, and DNA, which in turn impacts mitochondrial function. Post-mitotic cells, such as neurons, are highly susceptible to oxidative damage and mitochondrial dysfunction because they primarily rely on the OxPhos system to meet their substantial energy demands. At the cellular level, the activity of the respiratory chain seems to reduce with increasing age, thus increasing electron leakage and decreasing ATP production [21]. The cell produces less energy, and at the same time levels of oxidative stress rise, causing damage to other cellular metabolites [4,22]. Of note, ROS have also been shown to extend longevity by acting as a signalling molecule, according to the concept of “mitohormesis” or “oxidative eustress” [23]. This theory suggests that mild mitochondrial stress could protect the cells against subsequent perturbations. When reactive oxygen species (ROS) are produced in excess and overpower the cell’s antioxidant defenses, a condition known as oxidative distress occurs [24]. This can result in the damage of proteins, lipids, and DNA, leading to impaired cellular functions, accelerated aging, and an increased risk of diseases such as cancer, neurodegenerative disorders, and cardiovascular diseases. Interestingly, low levels of ROS were implicated in maintaining the quiescent state of hematopoietic stem cells (HSCs), while increases in ROS serve as signalling molecules to support stem cell differentiation [25]. Excessive levels of ROS were shown to create an oxidative microenvironment in pathophysiological states, such as aging leading to cellular damage [25]. 

Animal studies and human data have highlighted the age-related decrease in mitochondrial bioenergetics and antioxidant defenses and the increase in oxidative damage [4]. In 2014, a first study from Masotti and colleagues investigated the ‘biological aging’ of iPSCs, specifically using iPSCs derived from skin fibroblasts of a healthy individual [26]. The cells were kept in culture for over a year to simulate “aged” iPSCs, and for one month to represent “young” iPSCs. The “aged” iPSCs showed a decrease in MMP levels. Additionally, Petrini and colleagues suggested that iPSCs cultivated for over a year could serve as a new model for studying “premature” aging by examining the organization and expression patterns of major nuclear envelope constituents [27]. However, investigations at the mitochondrial level were missing. Interestingly, Birnbaum and colleagues showed aberrant mitochondrial function in iPSC-derived neurons from sporadic Alzheimer patients [28]. Namely, they observed that patient-derived cells produced more ROS and displayed changes in OxPhos chain complexes and higher levels of DNA damage. Similarly, in fibroblasts directly converted to neurons (induced neurons or iNs) from aged donors, mitochondria displayed a decrease in OxPhos-related gene expression, abnormal mitochondrial morphologies in the axons, lower MMP, a reduction in energy production, and an increase in protein oxidation [29]. These results align with our findings, particularly emphasizing the critical role of mitochondrial function in aging in advanced human cellular systems.

Because of the energy demand of the iPSCs to maintain cellular functions, we investigated the mitochondrial respiration and glycolysis in human iPSCs from young and aged donors. Our research findings indicate that induced pluripotent stem cells (iPSCs) obtained from older donors exhibit lower mitochondrial respiration than iPSCs from younger donors. Importantly, our study is the first to evaluate the glycolytic parameter in human iPSCs from aged donors, revealing a metabolic shift to glycolysis in these cells compared to iPSCs from younger donors. Indeed, we showed that human iPSCs from old donors increased their glycolytic activity, probably as a compensatory mechanism for the reduction in OxPhos. In fact, iPSCs are proliferative cells and ensure self-renewal and pluripotency processes through both glycolysis and OxPhos [30]. The metabolic shift from OxPhos to glycolysis in aged iPSCs can be explained by the Warburg effect, which is crucial for maintaining stem cell characteristics [30,31,32,33].

These data are consistent with another study from our group, where we investigated the aging phenotype of mitochondria in fibroblasts from the same young and aged donors as in the present report [34]. We observed decreased ATP and MMP levels, reduced mitochondrial respiration, an increase in ROS, and higher glycolytic activity compared to cells from young donors. Interestingly, older fibroblasts also showed a more fragmented mitochondrial network compared to younger fibroblasts. These findings suggest that the age-related mitochondrial impairments seen in fibroblasts from elderly donors are also present in their corresponding iPSCs, as shown in the present study. Therefore, iPSCs retain the mitochondrial aging characteristics from the donors. In the same study [34], these iPSCs were differentiated into neurons, and mitochondria bioenergetic impairments were observed in cells from aged donors. Also, the analysis of mitochondria-related genes revealed age-associated changes. Namely, the glutathione peroxidase (*gpx1*) expression involved in the antioxidant defenses was upregulated in iPSC-derived neurons from aged donors. In contrast, the expression of the uncoupling protein 2 (*ucp2*), which uncouples OxPhos from ATP synthesis, was downregulated. Genes involved in mitochondria fusion-fission (*mfn2*, *mfn1*, *fis1*, *drp1*, *opa1*) were unchanged in iPSC-derived neurons from aged donors compared to young. Further research is now required to complete these findings, especially about other mitochondrial functions like biogenesis, mitophagy, and fusion/fission activity.

Additional investigations addressing sex differences may also be necessary to characterize the mitochondrial function in iPSCs from aged donors. Indeed, one limitation of our study is that we cannot completely exclude that sex differences influenced the data obtained in our analyses. When looking at data from each donor in the aged group (Figure 4), we did not observe a sex difference (the SF854 male line does not differ from the other lines). As discussed by others [28], in vitro differences are unlikely since iPSCs are all grown in the same culture medium and not exposed to male or female sex hormones. Therefore, we believe that if a sex difference exists, it is somewhat due to sex hormone exposure that is known to influence mitochondrial function [35].

The advantage of using iPSCs is that they can be maintained indefinitely with a high cell number, stored easily, and are suitable for screening experiments. Also, iPSCs can differentiate into other cell types, including neurons, making them a potential model that can be a useful tool for screening brain-targeting drugs. Previous reports have indicated that the use of iPSC technology in aging research may be limited by cellular rejuvenation, resulting in a reset of age markers associated with the source cells [36,37]. Especially, the process of inducing pluripotency appears to reset age-related gene expression [14,38]. Here, we demonstrate that aged iPSCs exhibit mitochondrial bioenergetic impairments similar to those found in the corresponding fibroblasts. Thus, it seems that iPSCs retain certain aging characteristics from the original donor, particularly in terms of mitochondrial bioenergetics.

In conclusion, our findings demonstrated that iPSCs from aged donors still show age-related changes in mitochondrial function, indicating signs of aging without complete rejuvenation. Specifically, aged iPSCs presented impaired mitochondrial bioenergetics, leading to respiratory failure and decreased ATP, along with a loss of MMP, increased production of mitochondrial reactive oxygen species, and changes in mitochondrial network morphology. These findings suggest that the aging signature is preserved in iPSCs from aged donors, providing an opportunity for drug screening to promote healthy aging and potentially to prevent or treat age-related diseases at the mitochondrial level.

## 4. Materials and Methods

### 4.1. Chemicals and Reagents

Perkin Elmer (Waltham, MA, USA) supplied the ATPlite 1-step luminescence assay. DUTSCHER (Bernolsheim, France) supplied phosphate-buffered saline (PBS). Invitrogen (Waltham, MA, USA) supplied MitoSOX™ Red Mitochondrial Superoxide Indicator. Gibco (Waltham, MA, USA) provided the TrypLE™ Select Enzyme. Tetramethylrhodamine methyl ester perchlorate (TMRM), Hanks’ Balanced Salt Solution (HBSS), and dihydrorhodamine 123 (DHR) were all were purchased from Sigma-Aldrich (St. Louis, MO, USA). Takara Bio (Kusatsu, Shiga, Japan) supplied the Cellartis DEF-CS 500 COAT-1 and the DEF-CS 500 culture system. Seahorse XF DMEM Medium, pH 7.4, Seahorse XF calibrant, Mito stress test kit, glutamine, glucose, and pyruvate were supplied from Agilent (Santa Clara, CA, USA).

### 4.2. Human iPSCs Culture Model

Takara, StemBANCC consortium, and Dr. Zameel Cader from the Oxford’s University kindly provided the iPSC lines from healthy adult donors (Table 1). Of note, all the cells used in this study are commercially available or from biorepositories. Therefore, they are excluded from the Human Research Act (HRA) and do not require ethical approval. Regarding aged donors, no disease was diagnosed at the time of the biopsy. They are, therefore, considered healthy.

Feeder-free conditions were used to maintain iPSCs. Plates were coated with DEF-CS COAT-1, and cells were grown using DEF-CS basal medium supplemented with GF-1 (1:333) and GF-2 (1:1000). To reduce the cell death after passing or plating, the medium was supplemented with the supplement GF-3 (1:1000). The cells were passaged once a week with TripLE Select. The culture medium was changed daily. Cells were incubated in a humidified incubator at 37 °C and 5% CO_2_.

### 4.3. ATP Levels

A bioluminescence assay was used to determine the total ATP content of iPSCs [39]. Bioluminescence represents the production of light by luciferase from ATP and luciferin. The Cytation 3 Cell Imaging Multi-mode Plate Reader (BioTek) was used to measure the emitted light, which was linearly related to the ATP concentration after blank subtraction corresponding to the condition with 0 μM ATP in the standard curve (no cells, only the cell culture medium and the ATPlite reagent). The iPSCs from young and aged donors were plated each at a density of 20,000 cells/well into coated white 96-well cell culture plate coated with COAT-1.

### 4.4. Determination of Mitochondrial Membrane Potential (MMP)

MMP was measured using the potentiometric fluorescent dye TMRM (tetramethylrhodamin-methylester). Cells were plated into a black 96-well cell culture plate coated with COAT-1 at a density of 20,000 cells/well. The next day iPSCs were incubated with TMRM (0.4 μM for 20 min). A blank condition, corresponding to well with cells but no dye, was used to subtract the cells’ autofluorescence from the fluorescence signal. Fluorescence was measured using the multiplate reader Cytation 3 (BioTek, Agilent, Santa Clara, CA, USA) at 531 nm (excitation)/595 nm (emission) after two washes with HBSS. The fluorescence intensity of the TMRM, was dependent on the MMP.

### 4.5. Detection of Reactive Oxygen Species Levels (ROS)

Fluorescent dyes DHR (dihydrorhodamine 123) and the red mitochondrial superoxide indicator (MitoSOX) were used to measure levels of mitochondrial ROS and mitochondrial superoxide anion radicals, respectively. The iPSCs were seeded at a density of 20,000 cells/well into black 96-well cell culture plates, COAT-1 coated. At room temperature, the following day, the iPSCs were incubated with DHR (10 μM for 15 min) or MitoSOX (5 μM for 2 h) in the dark on a shaker. A blank condition, corresponding to well with cells but no dye, was used to subtract the cells’ autofluorescence from the fluorescence signal. After two washes with HBSS, the green fluorescent product generated by the oxidation of DHR was detected at 485 nm (excitation)/538 nm (emission) using the Cytation 3 Cell Imaging Multi-mode Plate Reader (BioTek, Agilent, Santa Clara, CA, USA). At 531 nm (excitation)/595 nm (emission), MitoSOX shows a red fluorescence. Fluorescence intensity was proportional to mitochondrial ROS levels and superoxide anion levels.

### 4.6. Bioenergetic Phenotype

The Seahorse XF HS Mini Analyser (Agilent, Santa Clara, CA, USA) was used to investigate key parameters related to mitochondrial respiration and glycolytic function. The oxygen consumption rate (OCR, mitochondrial respiration) was first measured in real time separately from the extracellular acidification rate (ECAR, glycolysis). For both assays, cells (20,000 cells/well) were plated on Seahorse COAT-1 coated miniplates. The following day, for the OCR measurement, the XF Mito Stress Test protocol was carried out for the OCR measurement. The assay medium to measure the OCR consisted of the Seahorse XF DMEM medium at pH 7.4 and 18 mM glucose, 2 mM L-glutamine and 4 mM pyruvate. The glycolysis stress assay was performed for the ECAR measurement. The assay medium used to measure the ECAR was composed of the Seahorse XF DMEM medium, with a pH of 7.4 and supplemented with 2 mM L-glutamine and 4 mM pyruvate. The OCR values were measured after sequential injection of 1.5 µM oligomycin, 1 µM FCCP, and a mix of 0.5 µM antimycin A and 1 µM rotenone. The OCR and ECAR were measured separately under basal conditions. To investigate the glycolytic function, sequential compound injections were performed to measure glycolysis, glycolytic capacity, and a calculate of glycolytic reserve and non-glycolytic acidification. The ECAR values were recorded by sequential injection of 18 mM glucose, 2 μM oligomycin, and 25 mM 2-deoxy-glucose. Bioenergetic parameters, especially the basal respiration, were automatically calculated on the Agilent Seahorse Analytics website.

### 4.7. Evaluation of Mitochondrial Mass

The iPSCs (20,000 cells/well) were seeded in a 96-well plate COAT-1 coated. After 24 h, mitochondrial mass was determined using the dye MitoTracker Green FM (Waltham, MA, USA) (100 nM, 1 h). A blank condition, corresponding to well with cells but no dye, was used to subtract the cells’ autofluorescence from the fluorescence signal. Fluorescence was measured using the Cytation 3 Cell Imaging Multi-mode Plate Reader (BioTek, Agilent, Santa Clara, CA, USA) at 490 nm (excitation)/516 nm (emission).

### 4.8. Mitochondrial Network Morphology

The iPSCs were stained with the MitoTracker™ Red CMXRos to visualise and quantify the influence of aging on mitochondrial morphology. The iPSCs from young and aged donors (20,000 cells/well) were plated COAT-1-coated 96-well cell culture plates. For mitochondrial staining, cells were incubated with the MitoTracker™ Red CMXRos for 30 min at room temperature. Then, 2% formaldehyde was used for cell fixation (for 15 min at room temperature). Nikon Eclipse Ti2-E inverted microscope with an oil objective was used to capture images at a magnification of 94.5× (63× + 1.5×). The FIJI software (version 2.14.0, https://imagej.net/software/fiji/) was used to assess changes in mitochondrial shape as previously described [40]. The raw images were first processed using background subtraction with a rolling ball radius of 20 pixels. To address uneven mitochondrial labelling, local contrast enhancement was performed using Contrast Limited Adaptive Histogram Equalization (CLAHE). Mitochondria were then segmented using the “Tubeness” filter. The perimeter and area of the mitochondria were determined using automatic thresholding and the “Analyze Particles” command. To evaluate mitochondrial length, the “Skeletonize” function was applied.

The morphometry macro on the FIJI software generated average metrics including area, perimeter, major and minor axis of elliptical fit of binary particles, and area after skeletonizing the binary particles [40]. The following parameters were then calculated:

− Area2: this measures the average size of mitochondria;
− Form factor: this describes the particle’s shape complexity as the inverse of circularity and is particularly reliable for well-separated mitochondria. It is used as an indicator of mitochondrial elongation;
− Area-weighted form factor: this is a variant of the form factor, providing more credible results in cases where highly elongated mitochondria overlap;
− Aspect ratio: this is defined as the ratio of the major and minor axis, which is independent of the area and perimeter. It is used as an indicator of mitochondrial branching;
− Length: this reports the mitochondrial length in units of pixels after reducing mitochondria to single-pixel-wide shapes.

### 4.9. Data Normalization

All the data were normalized on the living cell area assessed using the CellTracker Blue dye (Invitrogen, Waltham, MA, USA) [41]. The stock solution of CellTracker Blue was prepared in DMSO at a concentration of 10 mM. The iPSCs were loaded with the dye at a final concentration of 5 µM and incubated in the dark for 30 min at 37 °C. After one wash with HBSS, the fluorescence signal was detected at 353 nm (excitation) and 466 nm (emission) using the Cytation 3 Cell Imaging Multi-mode Plate Reader from BioTek. Of note, the dye loading was performed before the ATP measurement, as the ATP assay implies lysing the cells. For all other experiments, the dye loading was conducted afterward. The quantified CellTracker Blue signal was used to normalize the obtained data from the different experiments.

### 4.10. Statistical Analysis

Graph Pad Prism 9 was used for statistical analysis. Statistical comparisons were made using the Student’s unpaired *t*-test between the iPSCs from young donors and the iPSCs from aged donors. Linear regressions were determined using Pearson’s correlation coefficients. Values are considered statistically significant if *p*-values < 0.05.

## Figures and Tables

**Table 1:** iPSC lines from healthy adult donors used in this study.

Cell Line Code	Identifier or Catalog Number	Sex	Age (Year)	Origin	Source	Donor Group
SF841	SF841	M	36	Human skin fibroblasts	Cader Laboratory	Young
ChIPSC12	Cellartis^®^ Human iPS Cell Line 12	M	24	Human skin fibroblasts	Takara Bio	
ChIPSC18	Cellartis^®^ Human iPS Cell Line 18	M	32	Human skin fibroblasts	Takara Bio	
ChIPSC22	Cellartis^®^ Human iPS Cell Line 22	M	32	Human skin fibroblasts	Takara Bio	
SF180	SF180	F	60	Human skin fibroblasts	Cader Laboratory	Old
SF854	SF854	M	72	Human skin fibroblasts	Cader Laboratory	
SF840	SF840	F	67	Human skin fibroblasts	Cader Laboratory	
SF856	SF856	F	78	Human skin fibroblasts	Cader Laboratory	

## Data Availability

The data presented in this study are available on request from the corresponding author.

## Supplementary Materials

The following supporting information can be downloaded at: https://www.mdpi.com/article/10.3390/ijms252011199/s1.
