# Supplementary material for: Human iPSCs from Aged Donors Retain Their Mitochondrial Aging Signature
Source: Int J Mol Sci. 2024 Oct 18;25(20):11199. doi: 10.3390/ijms252011199 (PMC11508692; doi:10.3390/ijms252011199)
Supplement: Supplementary file 1 [file ijms-25-11199-s001.zip › ijms-3252761-supplementary.pdf]

Supplementary figures and captions :

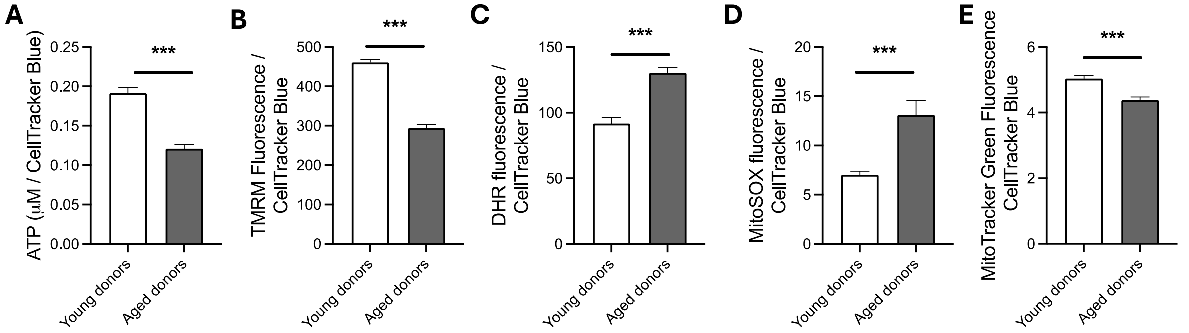

Supplementary Figure S1, related to Figure 1: Bioenergetic readouts in iPSCs from young versus old donors. (A) ATP levels were assessed using a bioluminescent assay (ATPlite). (B) Mitochondrial membrane potential (MMP) was assessed using the fluorescent dye tetramethylrhodamine methyl ester perchlorate (TMRM). (C) Mitochondrial reactive oxygen species levels were evaluated using the dihydrorhodamine 123 (DHR) fluorescent dye. (D) Mitochondrial superoxide anion levels were measured using the MitoSOX fluorescent dye. (E) Mitochondrial mass was evaluated using the MitoTracker Green fluorescent dye. All the data were normalized on the living cell area assessed using the CellTracker Blue fluorescence intensity. Data are presented as the mean ± SEM of three independent experiments (ATP: n=133-134 replicates, MMP: n=124-129 replicates, mROS: n=123-134 replicates, mSuperox.: n=124-131, Mito. Mass n=128-150 replicates per condition, four human iPSCs from young donors or four human iPSCs from aged donors per group). Student t-test between iPSCs from young donors vs iPSCs from aged donors ***P<0.001.

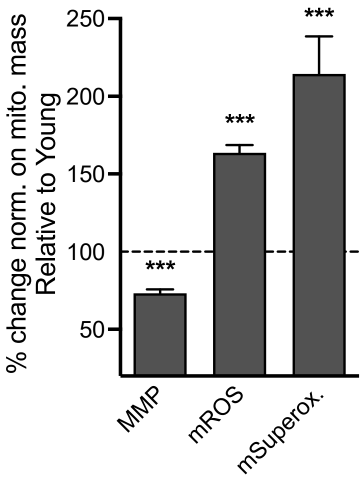

Supplementary Figure S2 : Bioenergetic readouts in iPSCs from young versus old donors normalized on mitochondrial mass. Readouts include mitochondrial membrane potential (MMP), mitochondrial ROS (DHR), and mitochondrial superoxide anion (MitoSOX) levels. Data were normalized on the Mitotracker green signal, an MMP-independent mitochondrial dye used to assess mitochondrial mass. Values are shown as the percentage of iPSCs derived from young donors and represent the mean ± SEM of three independent tests (n=120-130 replicates per condition, four human iPSCs from young donors or four human iPSCs from aged donors per group). Student *t-test* between iPSCs from young donors vs iPSCs from aged donors ***P<0.001.

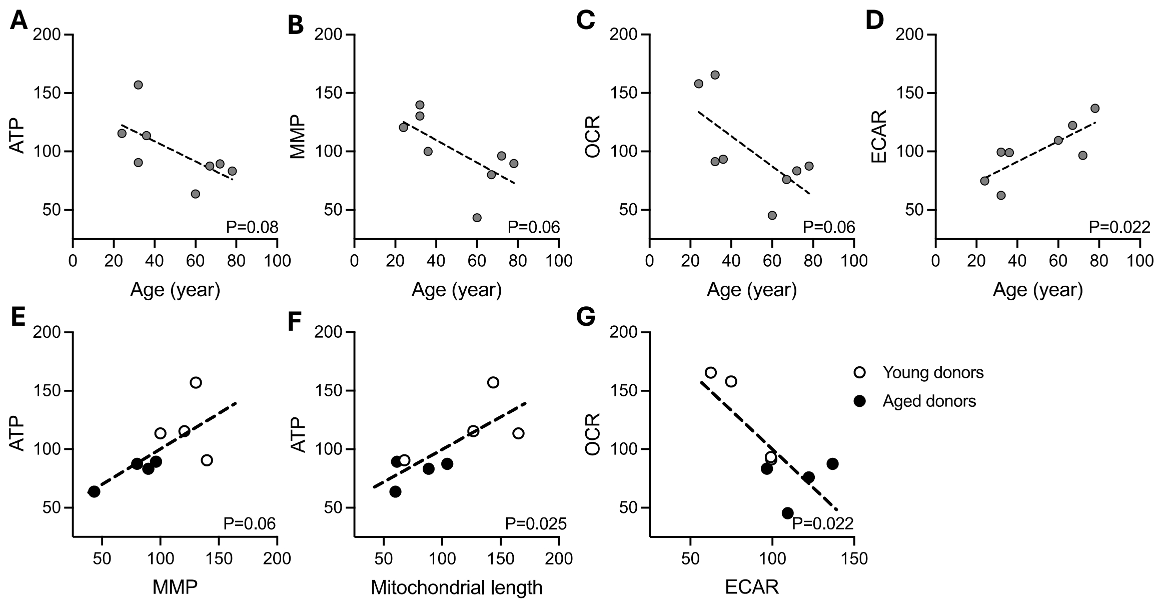

Supplementary Figure S3: Correlations between age and bioenergetic parameters in iPSCs from young and aged donors. Linear regression analyses were performed between (A) ATP level versus age, (B) mitochondrial membrane potential (MMP) versus age, (C) basal oxygen consumption rate (OCR) versus age, (D) basal extracellular acidification rate (ECAR) versus age, (E) ATP level versus MMP, (F) ATP level versus mitochondrial length, and (G) OCR versus ECAR in iPSCs from young and aged donors. Values represent the mean of each donor (n= 4 young and 4 aged) in the percentage of all donors. The p-values obtained with a simple linear regression test are indicated on each graph.
